# γH2AX, 53BP1 and Rad51 protein foci changes in mesenchymal stem cells during prolonged X-ray irradiation

**DOI:** 10.18632/oncotarget.19203

**Published:** 2017-07-12

**Authors:** Anastasia Tsvetkova, Ivan V. Ozerov, Margarita Pustovalova, Anna Grekhova, Petr Eremin, Natalia Vorobyeva, Ilya Eremin, Andrey Pulin, Vadim Zorin, Pavel Kopnin, Sergey Leonov, Alex Zhavoronkov, Dmitry Klokov, Andreyan N. Osipov

**Affiliations:** ^1^ Lomonosov Moscow State University, Moscow 119991, Russia; ^2^ State Research Center - Burnasyan Federal Medical Biophysical Center of Federal Medical Biological Agency (SRC-FMBC), Moscow 123098, Russia; ^3^ Insilico Medicine, Inc, ETC, Johns Hopkins University, Baltimore, Maryland 21218, USA; ^4^ Semenov Institute of Chemical Physics, Russian Academy of Sciences, Moscow 119991, Russia; ^5^ Emanuel Institute for Biochemical Physics, Russian Academy of Sciences, Moscow 119991, Russia; ^6^ Federal State Budgetary Institution “Central Clinical Hospital with Outpatient Health Center” of The Business Administration for The President of The Russian Federation, Moscow 121359, Russia; ^7^ PJSC Human Stem Cells Institute, Moscow 119333, Russia; ^8^ N.N. Blokhin Cancer Research Center, Moscow 115478, Russia; ^9^ Moscow Institute of Physics and Technology, Moscow 141700, Russia; ^10^ Canadian Nuclear Laboratories, Chalk River, Ontario K0J1P0, Canada

**Keywords:** DNA double strand breaks, DNA repair, mesenchymal stem cells, X-rays, continuous irradiation

## Abstract

At high exposure levels ionizing radiation is a carcinogen. Little is known about how human stem cells, which are known to contribute to tumorigenesis, respond to prolonged radiation exposures. We studied formation of DNA double strand breaks, accessed as γH2AX and 53BP1 foci, in human mesenchymal stem cells (MSCs) exposed to either acute (5400 mGy/h) or prolonged (270 mGy/h) X-irradiation. We show a linear γH2AX and 53BP1 dose response for acute exposures. In contrast, prolonged exposure resulted in a dose-response curve that had an initial linear portion followed by a plateau. Analysis of Rad51 foci, as a marker of homologous recombination, in cells exposed to prolonged irradiation revealed a threshold in a dose response. Using Ki67 as a marker of proliferating cells, we show no difference in the γH2AX distribution in proliferating vs. quiescent cells. However, Rad51 foci were found almost exclusively in proliferating cells. Concurrent increases in the fraction of S/G2 cells were detected in cells exposed to prolonged irradiation by scoring CENPF-positive cells. Our data suggest that prolonged exposure of MSCs to ionizing radiation leads to cell cycle redistribution and associated activation of homologous recombination. Also, proliferation status may significantly affect the biological outcome, since homologous repair is not activated in resting MSCs.

## INTRODUCTION

Nuclear accidents, such as those in Chernobyl and Fukushima, may result in exposure of humans to low and intermediate doses of ionizing radiation (IR) [1–3]. Even though, based on atomic bomb survivor studies, it is well established that high-dose exposures result in statistically significant increased risks of cancer and life shortening [4], whether low and intermediate doses can cause cancer or other biological detriment cannot be reliably determined from human studies due to their low statistical power [5]. Therefore, regulatory bodies have adopted a linear-no-threshold model which dictates that any excess dose of IR, however small, will result in an excess of cancer risk [6]. This model however has been widely criticized as not scientifically justified, since a multitude of studies have shown either the lack of biological detriment or even beneficial effects upon low-dose radiation exposures in a variety of experimental models [7–11]. This uncertainty about managing risks of low-dose radiation exposures also includes a dose-rate component, in that both dose and dose rate could affect the overall risk. Since most real-life scenarios of human exposures to low and intermediate doses of IR include prolonged exposures, lasting from hours to days, the dose-rate factor has been recognized as an important component of risk calculation. Indeed, the National Research Council and the National Academy of Sciences recommended a dose and dose rate effectiveness factor (DDREF) of 1.5 [12], whereas the International Committee on Radiological Protection (ICRP) suggested a DDREF of 2.0 [13]. The DDREF defines the fold-reduction in risk if a dose has been delivered chronically. However, more conservative estimates of prolonged vs. acute radiation exposure risks have been proposed [14, 15], indicating that there is a substantial controversy on how to regulate risks of exposure to prolonged vs. acute irradiation.

Systemic biological outcomes of human exposure to IR, such as cancer and shortening of life span, are the results of complex cellular and tissue responses to radiation. One of the main contributor to diseased conditions is damaged DNA [16]. As a result, a wealth of studies examined the biological effects of radiation exposure in experimental models which focused on DNA damage end-points, with a particular emphasis on DNA double-strand breaks (DSBs) or gross chromosomal changes that are directly caused by DSBs [17]. These lesions, if left unrepaired or misrepaired, can cause cell death [18] or lead to mutagenesis [19] and, consequently, to tumorogenesis [17]. Therefore, understanding responses of DNA DSB formation and repair followed by a particular radiation exposure mode is an important step toward understanding the potential biological consequence of such exposure.

Until recently, it has been commonly assumed that mutated somatic cells are the ones that give rise to tumor formation. However, this paradigm has shifted toward realization of a pivotal role that stem cells play in tumor initiation, progression and metastasis [20, 21]. Yet, our knowledge of DNA damage and repair responses to radiation comes predominantly from somatic cell models. Although human stem cells have been studied for their DNA damage and repair responses to acute radiation exposures [22–24], very few studies have used prolonged irradiation [25]. Multipotent mesenchymal stem cells (MSCs) are a well-characterized type of stem cells [26], widely used in stem cell-based therapy of various diseases [26]. They have a fibroblast-like phenotype and are able to renew themselves. Renewal and differentiation of MSCs is associated with a fine balance between proliferation and quiescence. How proliferating vs. resting MSCs respond to radiation is unknown. MSCs are considered to be relatively radioresistant in comparison to radiosensitive hematopoietic stem cells, mostly due to the activity of DNA DSBs repair pathways [27]. Therefore, MSCs can survive irradiation otherwise lethal to hematopoietic stem cells and can, in some cases, undergo neotransformation [28]. However, it is unknown how DNA DSBs are formed and repaired in these cells upon prolonged radiation exposures.

To address the knowledge gaps discussed above, we sought to study how DNA DSB responses are elicited in MSCs exposed to various doses of either acute (5400 mGy/h) or prolonged (270 mGy/h) X-radiation and how these responses are affected by their proliferation status. We used γH2AX and 53BP1foci as markers of DNA DSBs [29–31] and Rad51 foci as a marker of homologous recombination (HR) repair [32–34]. Both endpoints were measured in proliferating and quiescent MSCs using Ki67 as a marker of proliferating cells. Additionally, we measured the fraction of S/G2 cells to account for cell cycle redistribution and its potential contribution to responses to irradiation.

All experiments were performed using gingiva derived MSCs. Gingival mucosa is one of the most promising sources of MSCs due to its availability, low invasiveness of their collection procedure, and the ability of gingival mucosa wounds to heal without scarring [35]. In addition, gingiva derived MSCs may be more clinically valuable than MSCs derived from other tissues due to their higher proliferation capacity [36].

## RESULTS

### γH2AX and 53BP1 foci formation after acute or prolonged irradiation

**Figure 1:**
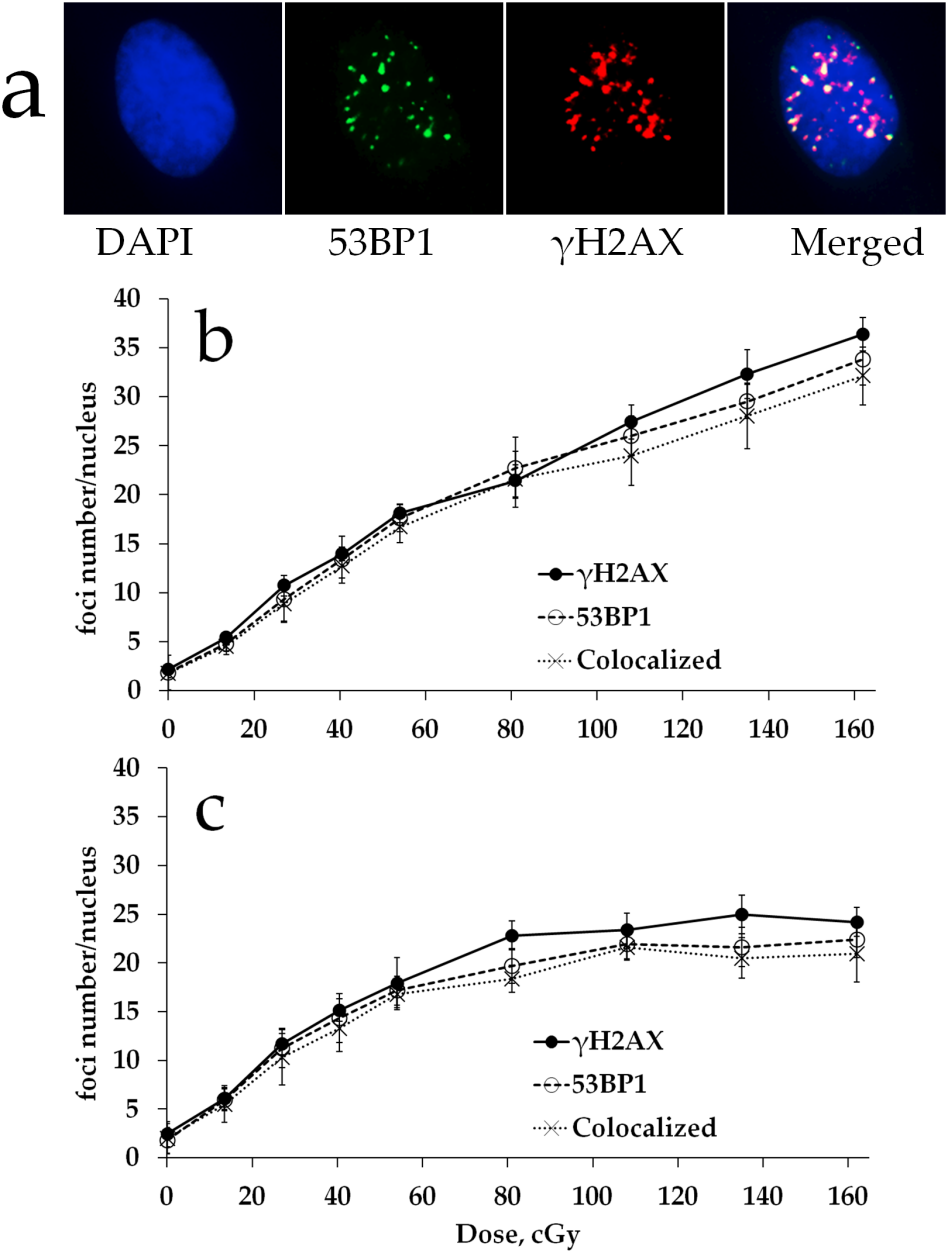
γH2AX and 53BP1 foci formation in MSCs exposed to either acute or prolonged X-ray irradiation **(a)** Representative microphotographs of immunofluorescently stained irradiated MSCs showing γH2AX (red) and 53BP1 (green) foci. DAPI counterstaining is shown in blue. **(b)** Quantification of γH2AX and 53BP1 foci, as well as their colocalization,in MSC exposed to acute (5400 mGy/h) or prolonged (270 mGy/h) **(c)** X-ray irradiation. Mean foci numbers derived from at least three independent experiments are shown. Error bars show SE.

We first characterized the formation of DNA DSBs in MSCs exposed to various doses of either acute (5400 mGy/h) or prolonged (270 mGy/h) X-ray irradiation by quantifying γH2AX and 53BP1 foci. A typical γH2AX and 53BP1 foci appearance pattern is shown in Figure 1a. Results of γH2AX and 53BP1 foci quantification are presented in Figure 1b, 1c. All radiation exposures produced statistically significant increases in γH2AX and 53BP1 foci numbers compared to the non-irradiated control. For high dose rate, the number of γH2AX and 53BP1 foci increased linearly with dose up to 1620 mGy (Figure 1b). This dose-response relationship was fit by a linear regression: *y=0.021x + 3.969* (R^2^=0.98), where *y* is a number of γH2AX foci and *x* is radiation dose in mGy. This result was consistent with our previous observations showing linear γH2AX dose responses in human fibroblasts [37], as well as with the results reported by others for this cell type [31]. Similar results were obtained for 53BP1 foci, another marker frequently used for quantification of DNA DSBs (Figure 1b). For prolonged irradiation, a different dose-response relationship was observed in that the initial linear portion of the curve turned into a plateau at around 1 Gy (Figure 1c). A statistically significant difference between acute and prolonged irradiation was found for doses of 1350 mGy (for γH2AX, p=0.0082; for 53BP1, p=0.0417) and 1620 mGy (for γH2AX, p=0.0009; for 53BP1, p=0.0229).

### Rad51 foci formation during prolonged irradiation

**Figure 2:**
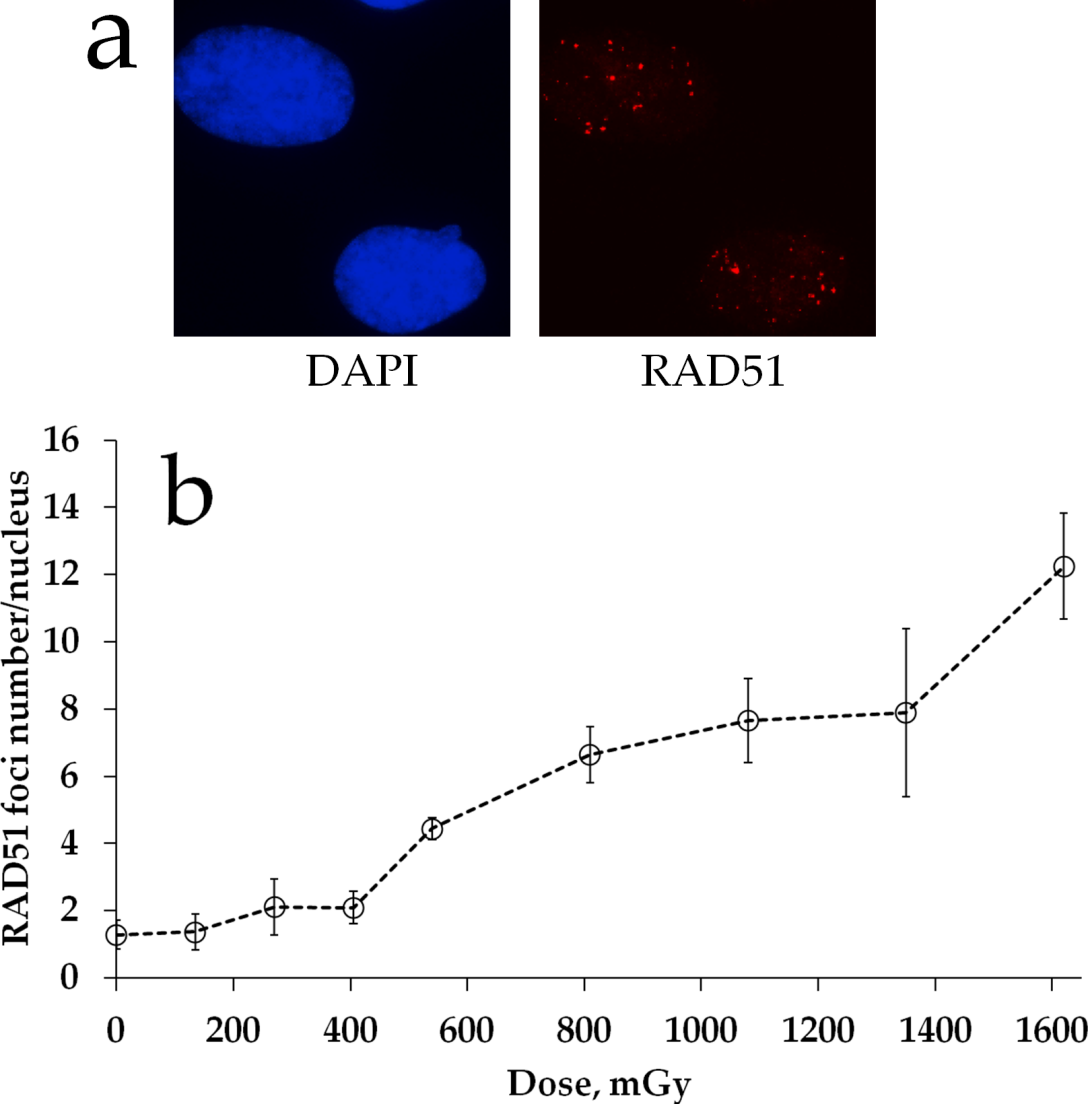
RAD51 foci formation in MSCs exposed to prolonged X-ray irradiation **(a)** Representative microphotographs of immunofluorescently stained irradiated MSCs showing Rad51 foci (red). DAPI counterstaining is shown in blue. **(b)** Quantification of Rad51 in MSC exposed to prolonged (270 mGy/h) X-ray irradiation. Mean foci numbers derived from at least three independent experiments are shown. Error bars show SE.

We examined the status of homologous DNA repair by quantifying Rad51 foci in cells exposed to prolonged X-ray irradiation. Figure 2a shows representative images of Rad51 foci in MSCs exposed to irradiation. Quantification of Rad51 foci is presented in Figure 2b. In contrast to γH2AX foci dose responses (Figure 1b), substantial increases in Rad51 foci were not found until about 2 h of prolonged irradiation (cumulative dose of 540 mGy). This finding suggests a threshold for homologous repair activation upon prolonged 270 mGy/h X-ray irradiation of MSC cultures. Between 2 and 6 h of irradiation, Rad51 foci accumulated linearly and the overall dose response could be fit by a linear regression *y=0.007x + 0.559* (R^2=^0.95), where *y* is a number of RAD51 foci and *x* is radiation dose in mGy. There was a dose overlap between the linear portion of Rad51 foci dose-response curve and the plateau portion of the γH2AX foci curve, suggesting that linear activation of homologous DNA repair may explain the plateau.

### γH2AX foci formation in Ki67+ vs. Ki67- cell subpopulations during prolonged irradiation

**Figure 3:**
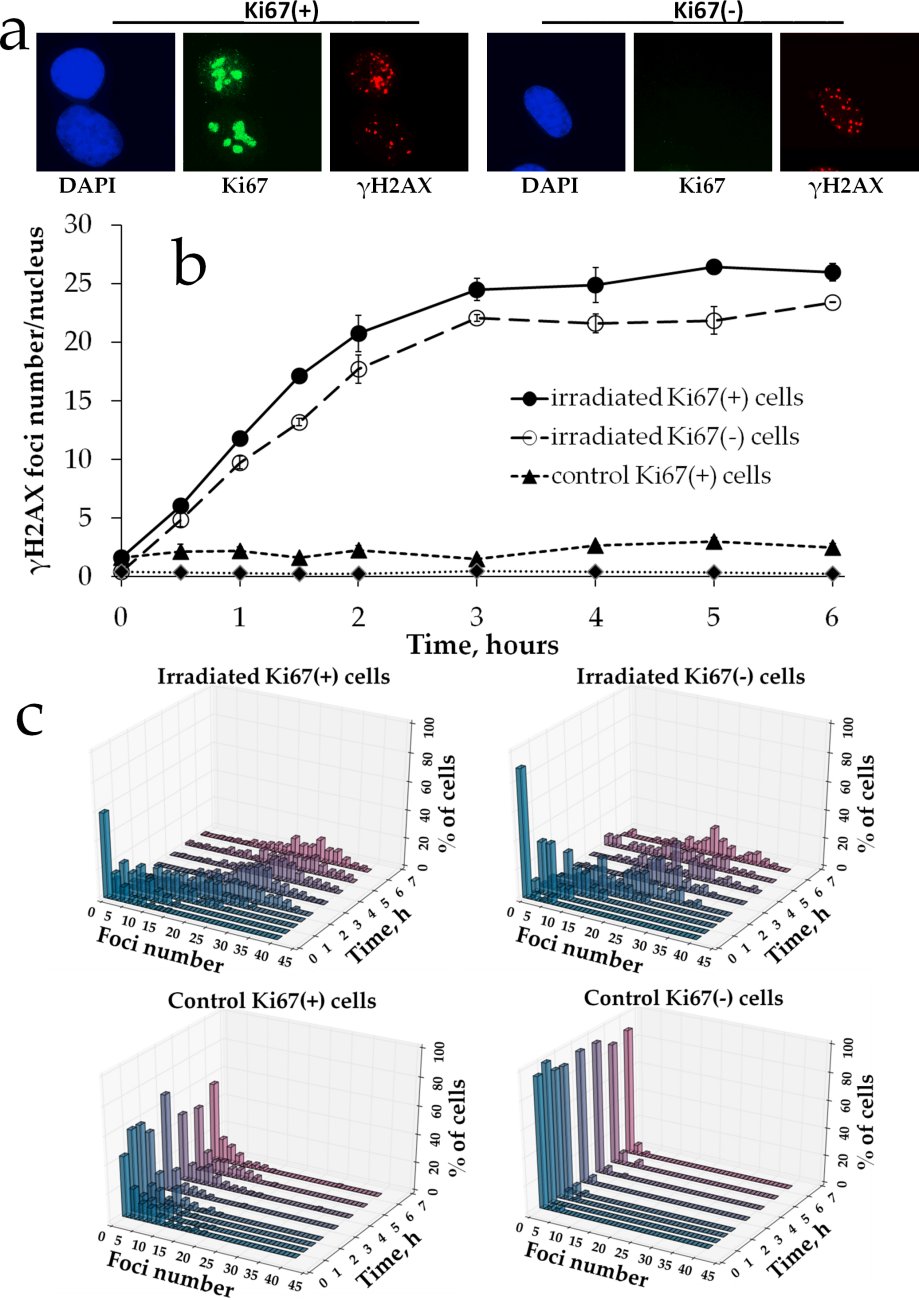
γH2AX foci formation in proliferating vs. resting MSCs exposed to prolonged X-ray irradiation **(a)** Representative microphotographs of immunofluorescently stained irradiated MSCs showing Ki67 (green) and γH2AX foci (red). DAPI counterstaining is shown in blue. **(b)** Quantification of γH2AX in Ki67+ vs Ki67- MSCs exposed to prolonged (270 mGy/h) X-ray irradiation. Mean foci numbers derived from at least three independent experiments are shown. Error bars show SE. **(c)** Histograms showing percent of cells with a certain number of γH2AX foci.

To further characterize γH2AX foci formation upon prolonged irradiation, we measured the responses in proliferating vs. non-proliferating cells. We used Ki67 as a marker of the proliferation status and scored γH2AX foci in Ki67 negative (Ki67-) G0 cells vs. Ki67 positive (Ki67+) interphase and mitotic cells (Figure 3a). First, we observed a statistically significant difference between the two subpopulations of control non-irradiated cells for each time point: 2.29 ± 0.36 for Ki67+ vs. 0.35 ± 0.08 for Ki67- cells (Figure 3b). Similarly, for irradiated cells for all of the time points examined the number of γH2AX foci was higher for Ki67+ subpopulation compared to Ki67- cells. We also constructed γH2AX histograms for each time point for these two subpopulations (Figure 3c) to examine heterogeneity of cells for γH2AX foci numbers. This data indicates that proliferating cells tend to have higher numbers of γH2AX foci. However, the shape of the dose-response curves did not differ between Ki67+ and Ki67- cells in that the plateau portion was evident for both and spanned the same dose range.

### Rad51 foci formation in Ki67+ vs. Ki67- subpopulations after prolonged irradiation

**Figure 4:**
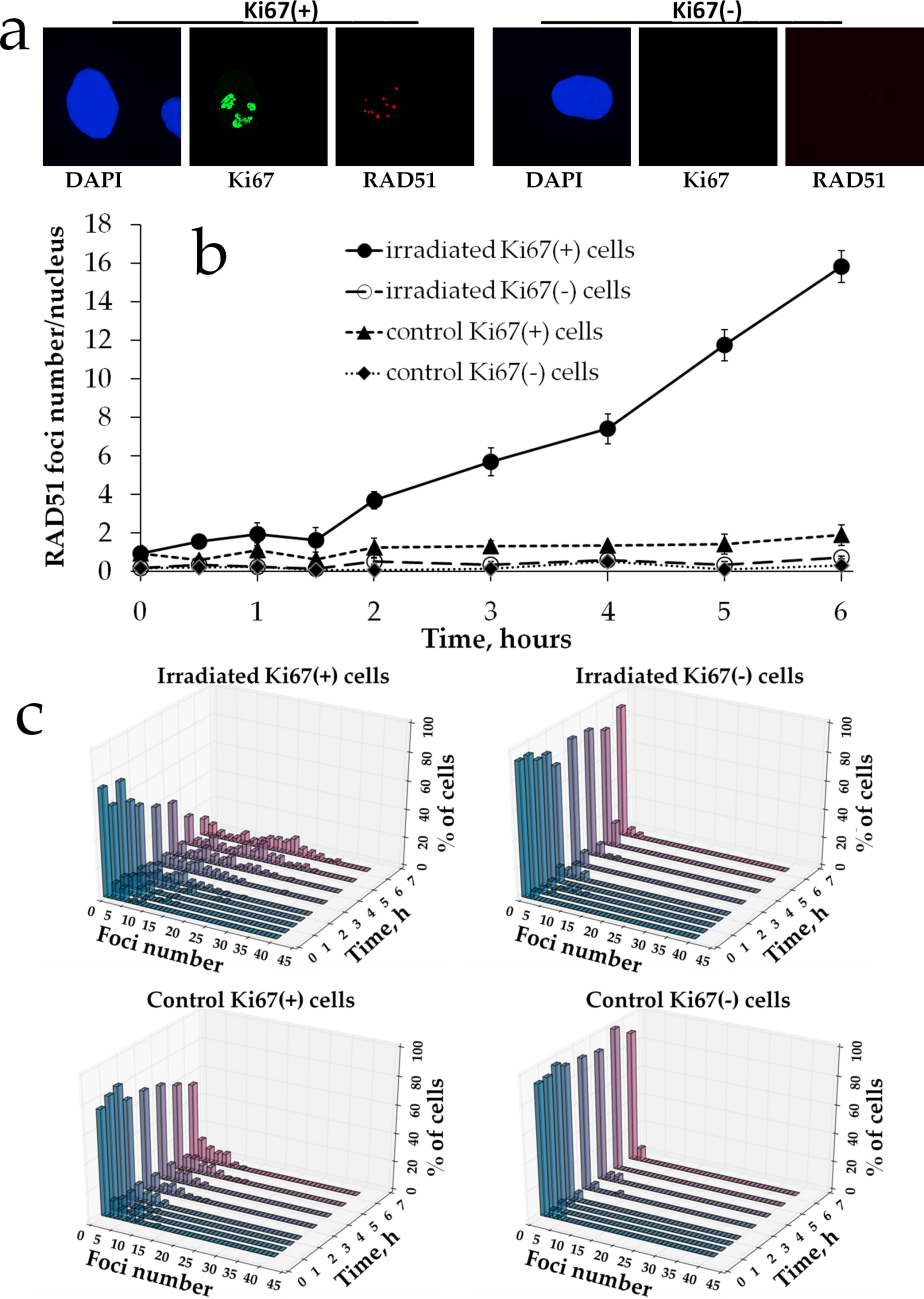
Rad51 foci formation in proliferating vs. resting MSCs exposed to prolonged X-ray irradiation **(a)** Representative microphotographs of immunofluorescently stained irradiated MSCs showing Ki67 (green) and Rad51 foci (red). DAPI counterstaining is shown in blue. **(b)** Quantification of Rad51 in Ki67+ vs Ki67- MSCs exposed to prolonged (270 mGy/h) X-ray irradiation. Mean foci numbers derived from at least three independent experiments are shown. Error bars show SE. **(c)** Histograms showing percent of cells with a certain number of Rad51 foci.

Next, we quantified Rad51 foci formation in proliferating vs. quiescent MSCs. Rad51 foci were not observed in Ki67- non-irradiated cells (mean value of 0.24 ± 0.12 foci per cell) as shown in a representative image in Figure 4b. Also, very few foci were found in Ki67+ control cells (1.23 ± 0.40 foci per cell). Interestingly, Rad51 foci were not produced in irradiated resting cells either and this lack of foci was found for all doses examined (Figure 4b). Only Ki67+ cells contained Rad51 foci upon irradiation. The dose response followed very closely the pattern seen for Rad51 foci for gross cell population in Figure 2b. Thus, foci did not start to accumulate until 1.5-2 h of the prolonged X-ray irradiation and the accumulation followed a linear pattern. The dose response was best fit (R^2^=0.99) by a linear-quadratic model *y=4.81x^2^+1.28x+1.09*, where *y* is a number of Rad51 foci and *x* is radiation dose in Gy. Finally, histograms showed that for all groups and time points distribution of cells with Rad51 was deviated from normality (Figure 4c).

### The fraction of proliferating cells did not change during the prolonged irradiation

**Figure 5:**
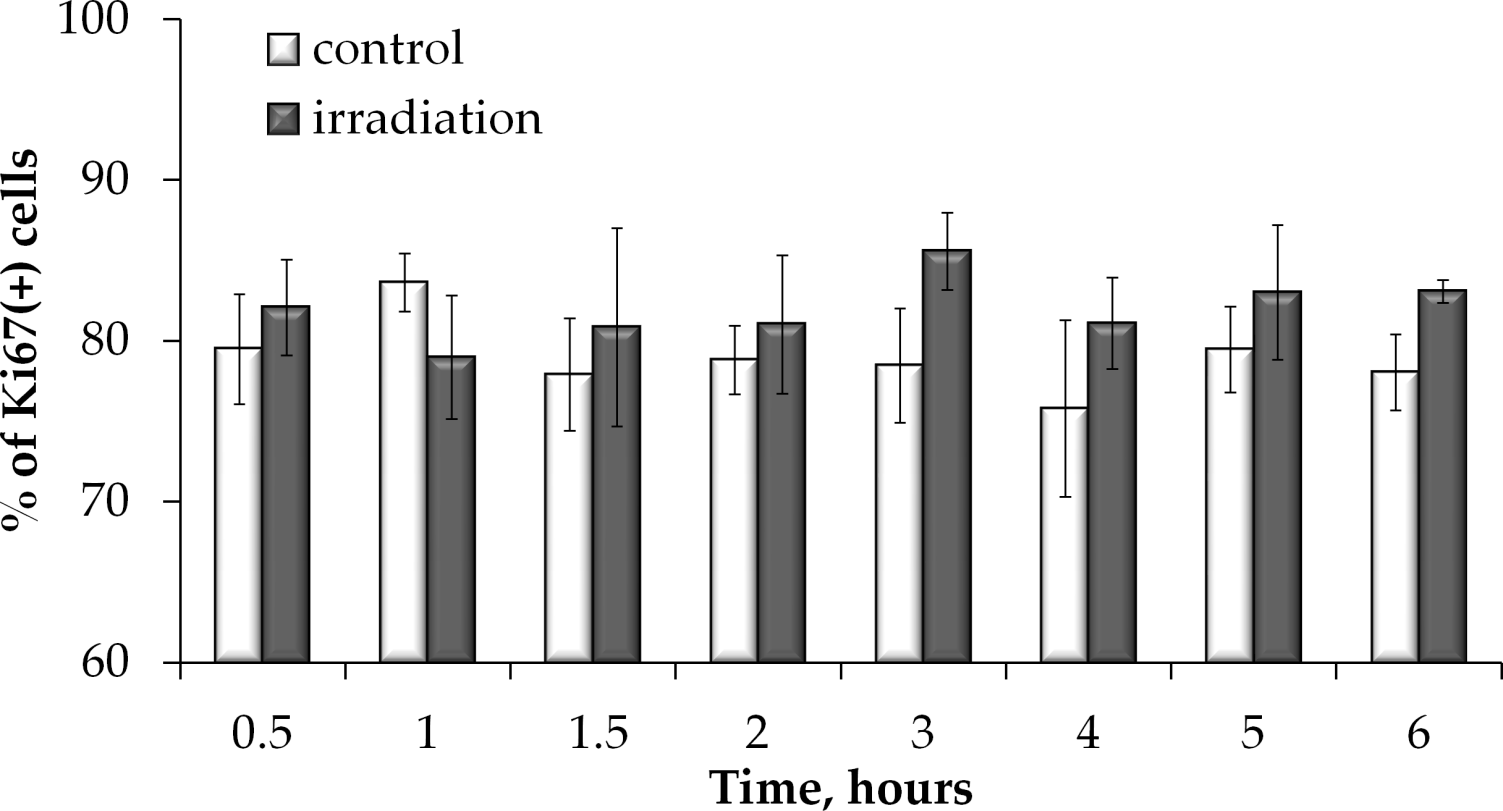
Proliferation is not affected by prolonged irradiation Ki67 positive cell were quantified in non-irradiated MSCs or cells exposed to prolonged X-ray irradiation and means of at least three independent experiments were plotted. Error bars show SE.

To examine whether the fraction of proliferating cells was affected by prolonged irradiation and may therefore have contributed to overall foci dose responses, we examined how the Ki67 positive cell fraction changed during prolonged irradiation. Results of this experiment presented in Figure 5 indicate that no statistically significant difference in the Ki67+ fraction in irradiated vs. non-irradiated cells was found. Throughout the study, the Ki67+ fraction averaged at ∼ 80 %. This data validates that there was no significant change in cell proliferation during prolonged irradiation and the responses obtained at various time points can be reliably compared to each other and to the control.

### The G2/M cell cycle arrest induction during the prolonged irradiation

**Figure 6:**
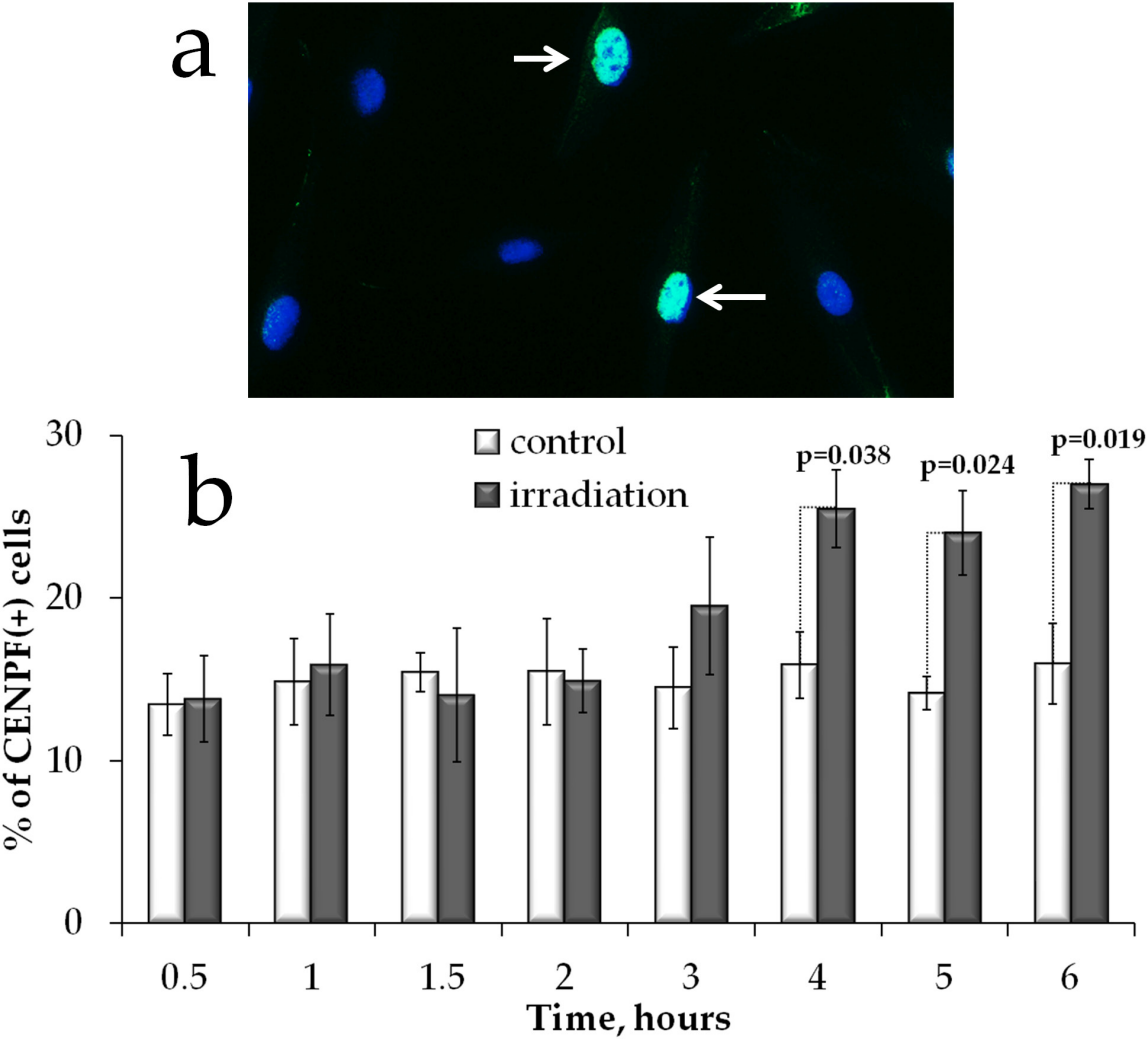
S/G2 cell cycle phases changes in MSCs exposed to prolonged irradiation **(a)** Representative microphotographs of immunofluorescently stained irradiated MSCs showing CENPF (green) DAPI counterstaining (blue). **(b)** Quantification of CENPF+ cells in cultures exposed to prolonged (270 mGy/h) X-ray irradiation. Mean values derived from at least three independent experiments are shown. Error bars show SE. p-values of statistically significant differences are shown.

HR DNA repair takes place only in S/G2 phases of the cell cycle when the sister chromatid is available to serve as a template. We therefore examined whether the increased Rad51 signal in proliferating cells could be attributed to accumulation of cells in S/G2 phases. We immunofluorescently labelled cells with CENPF and enumerated CENPF-expressing cells (CENPF+). This protein, being a component of the nuclear matrix during G2 phase, has been used as a marker of S/G2 cells [38, 39]. Its synthesis commences in early S phase and ceases in the M phase, with a peak in the G2 phase [39]. We determined that at 4-6 h of prolonged irradiation, a statistically significant (p<0.05) increase in the fraction of CENPF+ cells occurred (Figure 6b).

## DISCUSSION

The role of stem cells in radiation-induced carcinogenesis have long been overlooked. Relatively recent realization that stem cells substantially contribute to tumorigenesis, has led to a high interest in radiation responses in stem cells [40, 41]. Yet, a number of barriers, such as complexity of long-term biological consequences of radiation exposure and differences in the biology of stem cells of various types, exist in this rapidly developing area of radiation biology [42]. These barriers are behind substantial knowledge gaps that remain to be filled. One of them is the effect of dose rate on biological outcomes [43]. Results of the study by Shuryak et al. [44] showing that low LET IR directly causes normal mammary stem cells to acquire mutations *in vivo* implied that dose rate at low to intermediate dose is a substantial factor that may define an outcome. Our data showing that human MSCs exposed to prolonged X-irradiation accumulated γH2AX and 53BP1 foci differently compared to acute X-irradiation are consistent with such proposition.

Historically, plateau portions of dose-response curves have been well explained by inducible DNA repair [45, 46]. Similarly, we proposed that the plateau for γH2AX and 53BP1 foci formation observed between 800 and 1600 mGy for prolonged irradiation was due to inducible DNA repair that shifted a balance between newly formed and rejoined DNA DSBs towards the latter. Our previous report indicated that continuing irradiation tended to trigger HR repair in normal human fibroblasts [37]. For murine embryonic stem cells, Tichy et al. [47] found that HR, but not non-homologous end joining, was the DNA repair pathway of choice upon exposure to IR. When a dose response for Rad51 foci for prolonged X-irradiation was constructed for MSCs in this study, the results suggested that HR activation (between 560 and 1600 mGy) may have contributed to the plateau seen for γH2AX foci. However, this assumption was not confirmed when γH2AX and Rad51 foci were scored separately in proliferating and resting cells. It turned out that the pattern of the γH2AX foci dose response did not depend on the proliferation status of the cells, with the exception that slightly higher γH2AX foci numbers were observed in proliferating cells, which may represent an additional burden of transient metabolic foci (i.e. those related to DNA replication) (Figure 3). Interestingly, the difference in foci numbers between Ki67+ and Ki67- cells was similar for both irradiated and control cells, further supporting the notion of their relation to replication. Yet, a drastic difference was found between Rad51 dose response curves for proliferating vs. resting cells in that no foci were observed in resting cells (Figure 4). This data shows that the plateau portion of γH2AX/53BP1 dose response could not be attributed to inducible HR. It appears that a typical γH2AX decay associated with the completion of DSB rejoining with time (since time is another important parameter to keep in mind while considering prolonged irradiation) contributed to the formation of the plateau. Furthermore, out of two potential explanations of linear accumulation of Rad51 foci between 560 and 1600 mGy, one being HR activation and the other being accumulation of persistent DSBs, the latter deserves closer attention. Indeed, a complex relationship between repair and accumulation of DSBs was reported for quiescent and proliferating cells [48]. Minakawa et al. [48] showed that both resting and proliferating cells accumulate persistent DNA DSBs. While our data for Rad51 foci in replicating cells are consistent with this observation, the observed lack of foci in resting cells may be related to experimental differences, such as time of detection, doses and experimental model. Alternatively, it is still possible that HR repair is indeed activated in MSCs exposed to >560 mGy doses, however its contribution may not be detectable at the level of gross DNA DSBs provided by the γH2AX/53BP1 foci enumeration method. In this scenario, it could be anticipated that fewer mutations may be expected in MSCs exposed to prolonged vs. acute exposure, since HR is less prone to errors in break rejoining.

Low and intermediate doses of IR have been reported to affect proliferation of stem cells. Increased proliferation was found *in vitro* in rat MSCs exposed to 50 and 75 mGy [49], as well as *in vivo* in mice bone marrow hematopoietic progenitor cells irradiated with 75 mGy [50] and neural stem cells upon 300 mGy irradiation [51]. In this study we found that the proliferating fraction of cells measured as Ki67(+) cells did not change during the course of the entire prolonged irradiation experiment, suggesting that the proliferation activity stimulation/inhibition was not a factor in the observed shapes of dose responses for both γH2AX/53BP1 and Rad51 foci formation. However, we did observe an accumulation of cells in S/G2 phases of the cell cycle upon the prolonged irradiation, indicating that the increases in the HR may be a result of cell cycle redistribution.

The drastic difference in Rad51 foci formation between proliferating and quiescent cells may provide grounds for further focused studies to elaborate biological relevance of such responses. In particular, various stressful physiological conditions, such as heavy physical exercise, chemical poisoning and others that may be experienced by humans in response to an exposure scenario of moderate doses of prolonged irradiation (e.g. nuclear accidents) may shift the balance of quiescent vs. proliferating stem cells and thus affect biological long-term outcomes. Further studies examining the complex DNA damage responses in stem cells within the proliferation vs. quiescence context as they relate to stem cell driven neoplastic transformation and tumorigenesis are warranted. Results of this study showing distinct Rad51 foci responses in resting vs. proliferating human MSCs represent preliminary knowledge upon which these future studies may be based.

## MATERIALS AND METHODS

### Human MSCs isolation, characterization and cultivation

MSCs were isolated from oral mucosa (gingiva) biopsy of 4 healthy volunteers (women 26–32 years old). All donors signed the informed consent before procedures. Ethics approval for the study was granted by the Russian Healthcare Regulation Authority and the Ethics Committee and Academic Council of the Central Clinical Hospital with Outpatient Health Center. Biopsy specimens were placed in Dulbecco's Modified Eagles Medium (DMEM; StemCell Technology, USA) supplemented with 5% Fetal Bovine Serum (FBS; Biological Industries, Israel), 1 g/L D-glucose, 200 U/mL penicillin and 200 ug/mL streptomycin and transported to the laboratory. Samples of tissue were minced with sterile scalpels. Homogenates were then incubated with 1 mL of 0.25% Trypsin/EDTA (StemCell Technology, USA) at 37°C for 1 hour. Enzyme activity was blocked by adding 1 mL FBS (Biological Industries, Israel). Homogenates were centrifuged 7 minutes at 300 g. Obtained suspensions were incubated in 1 ml of 0.15% collagenase type II (Sigma, USA) at 37°C for 2 hours. Ten ml phosphate buffered saline (PBS, StemCell Technology, USA) was added and suspensions were centrifuged 7 minutes at 300 g and pellets resuspended in MesenCult medium (StemCell Technology, USA). Cells were seeded in culture flasks at the density of 3 × 105 cells/cm^2^ and incubated at 37°C and 5% CO_2_.

Differentiation of gingiva derived MSCs into the chondrogenic, adipogenic and osteogenic directions was performed using Human Mesenchymal Stem Cell Functional Identification Kit (R D Systems, USA) according to the manufacturer's procedure. Expression of osteocalcin, FABP4 and aggrecan was detected by immunocytochemical analysis using primary monoclonal antibodies against human osteocalcin, FABP4 and aggrecan provided in kit (R D Systems) and secondary fluorescein-labeled (FITC) antibodies (LifeTechnologies). All MSCs cultures maintained the ability to differentiate in osteogenic, adipogenic, and chondrogenic lineages when cultured in media supplemented with the corresponding induction factors. Immunofluorescence analysis showed expression of chondro- (aggrecan), osteo- (osteocalcin) and adipogenic (FABP4) markers after induction of differentiation. Expression of specific markers of differentiation was not observed when culturing gingiva derived MSCs under normal conditions.

For immunophenotypic characterization, cells at passage 2 were detached using 0.05% Trypsin/EDTA (StemCell Technology, USA), washed and counted. Ten to twenty thousand cells in PBSwere stained with labeled antibodies according to the manufacturer's instructions. Phenotyping was performed on a flow cytometer BD FACS Canto II (USA). For the identification and characterization of the cultured cells, the following set of monoclonal antibodies was used: CD90, CD73, CD105, CD54, CD44, CD13, CD34, CD117, CD45, CD14 (all from BD Bioscience, USA). The antibodies detect typical markers of mesenchymal progenitor cells. FITC-, APC-, PerCp- and PE-labeled IgG antibodies of corresponding class were used as isotype controls. Gingiva derived MSCs had a high level of expression of CD44, CD13, CD90, CD105, CD73, did not express markers of progenitor hematopoietic (CD34, CD45, CD14, CD117) cells, and had a low level of expression of adhesion molecule (CD54).

For irradiation experiments cells of the 2nd passage were used. Cells were detached, washed and resuspended and seeded at the density of 5 × 10^3^ cells/cm^2^ in 500 μL of culture medium onto coverslips (SPL Lifesciences, South Korea) placed inside 35 mm Petri dishes (Corning, USA). Additional volume of the medium (1,5 ml) was added after seeding. Cells were incubated at 37°C and 5% CO_2_ for at least 20 h before irradiation experiments.

### Irradiation

Cells were exposed to 100 kV X-rays at a dose rate of 270 mGy/h (0.8 mA, 1.5 mm A1 filter) and 5400 mGy/h using RUB RUST-M1 X-irradiator (Russia). Throughout the irradiation, cells were maintained at 37°C using thermo-granules Lab Armour (Life Technologies, USA). Cells were returned to normal growth conditions immediately after irradiation and maintained for various periods of time before fixation.

### Immunoflourescence staining

Cells were fixed on coverslips in 4% paraformaldehyde in PBS (pH 7.4) for 20 min for γH2AX staining and for 10 min for Rad51 staining at room temperature followed by two rinses in PBS and permeabilization in 0.3% Triton-X100 (in PBS, pH 7.4) supplemented with 2% bovine serum albumin (BSA) to block non-specific antibody binding. Cells were incubated for 1 hour at room temperature with primary antibody against γH2AX (dilution 1:200, clone EP854(2)Y, Merck-Millipore, USA), 53BP1 (dilution 1:200, clone BP13, Merck-Millipore, USA), RAD51 (dilution 1:200, ABE257, Merck-Millipore, USA), CENPF (dilution dilution 1:200, ab5, Abcam, USA) and Ki67 (dilution 1:200; clone Ki-S5, Merck-Millipore, USA) diluted in PBS with 1% BSA. After several rinses with PBS cells were incubated for 1 hour with secondary antibodies IgG (H+L) goat anti-mouse (Alexa Fluor 488 conjugated, dilution 1:600; Merck-Millipore, USA) and goat anti-rabbit (rhodamine conjugated, dilution 1:400; Merck-Millipore, USA) diluted in PBS (pH 7.4) with 1% BSA. Coverslips were then rinsed several times with PBS and mounted on microscope slides with ProLong Gold medium (Life Technologies, USA) with DAPI for DNA counter-staining. Cells were viewed and imaged using Nikon Eclipse Ni-U microscope (Nikon, Japan) equipped with a high definition camera ProgRes MFcool (Jenoptik AG, Germany). Filter sets used were UV-2E/C (340–380 nm excitation and 435–485 nm emission), B-2E/C (465–495 nm excitation and 515–555 nm emission) and Y-2E/C (540–580 nm excitation and 600–660 nm emission). 300-400 cells were imaged for each data point. Number of foci was counted using DARFI (https://github.com/varnivey/darfi).

### Statistical analysis

Statistical and mathematical analyses of the data were conducted using the Statistica 8.0 software (StatSoft). The results are presented as means of three independent experiments ± standard error. Statistical significance was tested using the Student t-test.
